# Post-marketing surveillance study on influenza vaccine in South Korea using a nationwide spontaneous reporting database with multiple data mining methods

**DOI:** 10.1038/s41598-022-21986-8

**Published:** 2022-11-24

**Authors:** Hyesung Lee, Bin Hong, SangHee Kim, Ju Hwan Kim, Nam-Kyong Choi, Sun-Young Jung, Ju-Young Shin

**Affiliations:** 1 School of Pharmacy, Sungkyunkwan University, 2066 Seobu-ro, Jangan-gu, Suwon-si, Gyeonggi-do 16419 South Korea; 2 Department of Biohealth Regulatory Science, Sungkyunkwan University, Suwon, South Korea; 3 Department of Health Convergence, Ewha Womans University, Seoul, South Korea; 4 Graduate School of Industrial Pharmaceutical Science, Ewha Womans University, Seoul, Korea; 5 College of Pharmacy, Chung-Ang University, Seoul, Republic of Korea; 6 Department of Global Innovative Drugs, Graduate School of Chung-Ang University, College of Pharmacy, Chung-Ang University, 84 Heukseok-ro, Dongjak-gu, Seoul, 06974 South Korea; 7 Department of Clinical Research Design & Evaluation, Samsung Advanced Institute for Health Sciences & Technology (SAIHST), Sungkyunkwan University, Seoul, South Korea; 8 Present Address: Department of Biohealth Regulatory Science, Sungkyunkwan University, Suwon, South Korea

**Keywords:** Vaccines, Influenza virus

## Abstract

Safety profiles of the influenza vaccine and its subtypes are still limited. We aimed to address this knowledge gap using multiple data mining methods and calculated performance measurements to evaluate the precision of different detection methods. We conducted a post-marketing surveillance study between 2005 and 2019 using the Korea Adverse Event Reporting System database. Three data mining methods were applied: (a) proportional reporting ratio, (b) information component, and (c) tree-based scan statistics. We evaluated the performance of each method in comparison with the known adverse events (AEs) described in the labeling information. Compared to other vaccines, we identified 36 safety signals for the influenza vaccine, and 7 safety signals were unlabeled. In subtype-stratified analyses, application site disorders were reported more frequently with quadrivalent and cell-based vaccines, while a wide range of AEs were noted for trivalent and egg-based vaccines. Tree-based scan statistics showed well-balanced performance. Among the detected signals of influenza vaccines, narcolepsy requires special attention. A wider range of AEs were detected as signals for trivalent and egg-based vaccines. Although tree-based scan statistics showed balanced performance, complementary use of other techniques would be beneficial when large noise due to false positives is expected.

## Introduction

Influenza is an infectious disease associated with high morbidity and mortality globally^1,2^, and vaccination is the most effective means of eradication^3^. In Korea, influenza vaccines have been provided as part of the National Immunization Program (NIP), and eligible groups have continuously expanded since their introduction in 1997. Accordingly, the number of adverse events (AEs) following immunization for influenza has been steadily increasing according to the Korea Adverse Event Reporting System (KAERS)^4^. Safety profiles are a collection of adverse events^5^, including the information identified from clinical trials or a post-marketing phase and unidentified to date. Especially, unidentified information or safety signal represents “reported information on a possible causal relationship between an AE and a vaccine or drug, of which the relationship is unknown or incompletely evaluated previously”^6^. In particular, concerns regarding serious AEs of influenza vaccination have been continuously highlighted, including Guillain–Barré syndrome (GBS), febrile seizure, anaphylaxis, narcolepsy, and Bell’s palsy^7–12^. However, only a few studies on AEs associated with influenza vaccines have been conducted in Korea^7,8^. Therefore, it is necessary to re-evaluate the safety information of influenza vaccines using the latest and widely collected Korean databases.

Influenza vaccines can be classified into two types in terms of the type of production method or virus immunization: (a) egg-based vaccines and cell-based vaccines for the former, and (b) trivalent influenza vaccines (TIVs) and quadrivalent influenza vaccines (QIVs) for the latter. Several studies have reported that the safety of influenza vaccines may vary according to subtypes^13–15^, but most of the evidence is dependent on the results of randomized clinical trials (RCTs) with a limited population and study period. Therefore, an additional study with a large-scale database is needed to establish safety profiles by subtype.

There are two types of methods to detect safety signals (quantitative and qualitative method), and a variety of quantitative data mining methods are available for post-marketing safety monitoring. Typically, disproportionality-based methods are widely used to detect safety signals based on an imbalance between the reporting rate of specific AEs for a specific drug and that of specific AEs for other drugs. The proportional reporting ratio (PRR), reporting odds ratio (ROR), and information component (IC) are the most common measurements in disproportionality-based methods^16^. Recently, tree-based scan statistics (TSS) have been applied in the signal detection of vaccines^17,18^, which were proposed to analyze a database with a hierarchical tree structure with adjustment for multiple comparisons^19^. Although various data mining methods have been developed and widely used for detecting signals, inconsistent results have been reported using different methods^17,18^, implying that the use of multiple data mining methods should be considered simultaneously.

Given the limited safety profiles on influenza vaccine and inconsistent results by different data mining methods, we conducted a nationwide, post-marketing surveillance study to detect adverse events following influenza vaccination using three different data mining methods. Firstly, we identified unexpected AEs which are potentially related to influenza vaccination, particularly focusing on five serious AEs of special interest (GBS, febrile seizure, anaphylaxis, narcolepsy, and Bell’s palsy). Secondly, we aimed to provide comparative safety profiles by the subtype of influenza vaccine (type of production method: egg-based vs. cell-based; type of virus immunization: QIVs vs. TIVs). Lastly, we compared the results of signal detection from each data mining methods to evaluate their performance on predicting safety signals.

## Results

### General characteristics

The basic characteristics of influenza vaccine-related AEs compared with those of other vaccines are provided in Table 1. More AEs were reported in females than in males for both influenza vaccines and all other vaccines, accounting for 68.77% and 57.30%, respectively. The proportion of children below 24 months in the influenza vaccine group was higher than that of other vaccines, while that of individuals 19 years or older in the influenza vaccine group was lower than that of other vaccines. When stratified by route of administration, most of the characteristics between the influenza vaccine and other vaccines were similar, except for the origin and year of the report: most came from a pharmaceutical company for all other vaccines (74.71%), while pharmaceutical companies with regional pharmacovigilance centers (RPVC) for influenza vaccines (52.84% and 42.57%, respectively) and the proportion of serious influenza vaccine-related AEs (5.18%) were less than that of other vaccines (8.06%).

**Table 1:** Characteristics of adverse event reports following influenza vaccination and all other vaccines between 2005 and 2019.

Characteristics	Total N = 38,221 N (%)		Influenza vaccine N = 17,378 N (%)		All other vaccines N = 20,843 N (%)		*P* value^α^
Sex							< 0.0001
Male	12,721	(33.28%)	5183	(29.83%)	7538	(36.17%)	
Female	23,892	(62.51%)	11,950	(68.77%)	11,942	(57.30%)	
Missing	1608	(4.21%)	245	(1.41%)	1363	(6.54%)	
Age group							< 0.0001
< 28 days	1275	(3.34%)	30	(0.17%)	1245	(5.97%)	
28 days to < 24 months	4401	(11.51%)	164	(0.94%)	4237	(20.33%)	
24 months to < 12 years	3736	(9.77%)	1808	(10.40%)	1928	(9.25%)	
12 years to < 19 years	2160	(5.65%)	1422	(8.18%)	738	(3.54%)	
19 years to < 65 years	12,676	(33.17%)	8037	(46.25%)	4639	(22.26%)	
65 years and above	2010	(5.26%)	1019	(5.86%)	991	(4.75%)	
Missing	11,963	(31.30%)	4898	(28.19%)	7065	(33.90%)	
Report type							< 0.0001
Spontaneous report	20,064	(52.49%)	8719	(50.17%)	11,345	(54.43%)	
Research (PMS included)	17,564	(45.95%)	8445	(48.60%)	9,119	(43.75%)	
Literature	269	(0.70%)	79	(0.45%)	190	(0.91%)	
Other	324	(0.85%)	135	(0.78%)	189	(0.91%)	
Original reporter							< 0.0001
HCPs (doctor, pharmacist, nurse)	25,639	(67.08%)	11,011	(63.36%)	14,628	(70.18%)	
Consumer	2763	(7.23%)	1105	(6.36%)	1658	(7.95%)	
Other (other HCPs, lawyer)	5991	(15.67%)	2147	(12.35%)	3844	(18.44%)	
Missing	3,828	(10.02%)	3115	(17.92%)	713	(3.42%)	
Report origin							< 0.0001
RPVC	9458	(24.75%)	7397	(42.57%)	2061	(9.89%)	
Pharmaceutical company	24,754	(64.77%)	9183	(52.84%)	15,571	(74.71%)	
Medical institute	378	(0.99%)	114	(0.66%)	264	(1.27%)	
Pharmacy	4	(0.01%)	1	(0.01%)	3	(0.01%)	
Public health center	62	(0.16%)	6	(0.03%)	56	(0.27%)	
Consumer	423	(1.11%)	30	(0.17%)	393	(1.89%)	
Other	3142	(8.22%)	647	(3.72%)	2495	(11.97%)	
Seriousness							< 0.0001
Yes	2581	(6.75%)	901	(5.18%)	1680	(8.06%)	
No	35,640	(93.25%)	16,477	(94.82%)	19,163	(91.94%)	

Abbreviations: HCPs, healthcare professionals; PMS, post-marketing surveillance; RPVC, regional pharmacovigilance center.

^α^*P* values indicate differences between influenza vaccine and all other vaccines. *P* < 0.05 was considered statistically significant.

### Signal detection

Compared to all other vaccines, we identified 36 AEs as safety signals of influenza vaccines detected by any data mining method at least once (see Supplementary Material S2 online). Among them, seven AEs (injection site inflammation, muscle weakness, quadriplegia, paraplegia, hyperventilation, cachexia, and narcolepsy) were not included in the labeling information and were detected by all data mining methods, except for paraplegia (not in TSS). For serious AEs of special interest, anaphylaxis, neuritis (a preferred term [PT] of GBS), and narcolepsy were detected as signals compared to all other vaccines by all data mining methods, and paralysis (a PT of Bell’s palsy) was detected by IC and TSS (Table 2). Additionally, each method detected a similar number of safety signals (PRR, 29; IC, 30; TSS, 27).

**Table 2:** Unlabeled and adverse events of special interest among signal detection results of influenza vaccination using disproportionality methods and tree-based scan statistics from 2005 to 2019.

Adverse events (WHO-ART PT code)	Frequency	PRR	IC	TSS	Signal detection			Labeling in MFDS
					PRR	IC	TSS	
**Special interest**								
**Body as a whole—general disorders**^†^								
Anaphylactic reaction	39	2.77	0.18	0.01	Y	Y	Y	Y
**Central & peripheral nervous system disorders**^†^								
Paralysis	107	1.96	0.20	0.002		Y	Y	Y
Neuritis	99	5.82	0.68	0.001	Y	Y	Y	Y
**Unlabeled**								
**Musculo-skeletal system disorders**^†^								
Muscle weakness	111	3.32	0.49	0.001	Y	Y	Y	N
**Application site disorders**^†^								
Injection site inflammation	1,429	2.99	0.69	0.001	Y	Y	Y	N
**Central & peripheral nervous system disorders**^†^								
Quadriplegia	32	5.46	0.39	0.001	Y	Y	Y	N
Paraplegia	24	2.92	0.05	0.266	Y	Y		N
**Respiratory system disorders**^†^								
Hyperventilation	18	10.23	0.32	0.002	Y	Y	Y	N
**Metabolic and nutritional disorders**^†^								
Cachexia	84	4.21	0.54	0.001	Y	Y	Y	N
**Special interest and unlabeled**								
**Psychiatric disorders**^†^								
Narcolepsy	11	18.75	0.16	0.011	Y	Y	Y	N

Abbreviations: WHO-ART, World Health Organization-Adverse Reactions Terminology; PT, preferred term; AE, adverse events; IC, information component; PRR, proportional reporting ratio; TSS, tree-based scan statistic; MFDS, Ministry of Food and Drug Safety of South Korea.

^†^Adverse events were categorized according to the WHO-ART System Organ Class.

Different findings have been reported for influenza vaccine subtypes using various methods. When using TIV as a comparator, 13 AEs were detected as safety signals by any data mining method at least once for QIV, and among them (see Supplementary Material S3 online), two safety signals (injection site bruising and cachexia) were not included in the labeling (Table 3). Moreover, each method generated a similar number of safety signals: 13 signals for PRR and TSS and 12 signals for IC. In contrast, we detected 59 AEs as safety signals at least once for TIV when compared to QIV; 26 safety signals were not included in labeling, but only 5 of these were overlapped by the three methods.

**Table 3:** Unlabeled signals among signal detection results of quadrivalent and trivalent influenza vaccines using disproportionality methods and tree-based scan statistics from 2005 to 2019.

Adverse events (WHO-ART PT code)	Frequency	PRR	IC	TSS	Signal detection			Labeling in MFDS
					PRR	IC	TSS	
**Unlabeled signals for TIV compared to QIV**								
**Application site disorders**^†^								
Injection site bruising	38	3.99	0.22	0.001	Y	Y	Y	N
**Metabolic and nutritional disorders**								
Cachexia	53	2.33	0.09	0.016	Y	Y	Y	N
**Unlabeled signals for TIV comparing to QIV**								
**Body as a whole—general disorders**^†^								
Tremor	48	11.73	0.13	0.001	Y	Y	Y	N
Hyperpyrexia	40	7.33	0.04	0.001	Y	Y	Y	N
Death	23			0.001			Y	N
Edema peripheral	19	2.79	0.39		Y			N
Edema mouth	13	4.76	0.46		Y			N
**Central & peripheral nervous system disorders**^†^								
Quadriplegia	31		0.07	0.001		Y	Y	N
Gait abnormal	31	7.57	0.04	0.001	Y		Y	N
Hypoesthesia	28	5.13	0.13	0.022	Y		Y	N
Dysphonia	19	2.79	0.39		Y			N
Stupor	14	10.26	0.34		Y			N
**Respiratory system disorders**^†^								
Pneumonia	93	4.01	0.15	0.001	Y	Y	Y	N
Hyperventilation	18			0.001			Y	N
Tracheitis	9	6.60	0.61		Y			N
**Skin and appendages disorders**^†^								
Rash erythematous	238	14.53	0.46	0.001	Y	Y	Y	N
Skin discoloration	12		0.03				Y	N
Bullous eruption	12	4.40	0.51		Y			N
**Gastro-intestinal system disorders**^†^								
Gastroenteritis	37	2.71	0.17		Y			N
Dyspepsia	31	2.27	0.28		Y			N
**Musculo-skeletal system disorders**^†^								
Muscle weakness	103	15.10	0.33	0.001	Y	Y	Y	N
**Resistance mechanism disorders**^†^								
Infection viral	30	3.66	0.16		Y			N
**Cardiovascular disorders**^†^								
Hypotension	20	14.66	0.15	0.009	Y		Y	N
Deafness	9	6.60	0.61		Y			N
**Hearing and vestibular disorders**^†^								
Vision abnormal	16	3.91	0.39		Y			N
**Reproductive disorders, female**^†^								
Dysmenorrhea	12	8.79	0.43		Y			N

Abbreviations: WHO-ART, World Health Organization-Adverse Reactions Terminology; PT, preferred term; AE, adverse events; IC, information component; PRR, proportional reporting ratio; TSS, tree-based scan statistic; MFDS, Ministry of Food and Drug Safety of South Korea; QIV, quadrivalent influenza vaccine; TIV, trivalent influenza vaccine.

^†^Adverse events were categorized according to the WHO-ART System Organ Class.

Compared to the egg-based vaccine, we identified 13 AEs as safety signals of cell-based vaccines using at least one data mining method (see Supplementary Material S4 online). Among them, four safety signals (injection site bruising, pneumonitis, encephalopathy, and tachycardia) were not included in the labeling, but only injection site bruising was detected by all three methods (Table 4). PRR, IC, and TSS detected 9, 13, and 6 safety signals, respectively. When using cell-based vaccines as a comparator, 30 AEs were detected as safety signals by any data mining method at least once for egg-based vaccines, 4 of which (leg pain, tremor, injection site mass, and cachexia) were not included in the labeling. PRR, IC, and TSS detected 20, 2, and 23 safety signals, respectively.

**Table 4:** Unlabeled signals among signal detection results of cell-based and egg-based influenza vaccines using disproportionality methods and tree-based scan statistics from 2005 to 2019.

Adverse events (WHO-ART PT code)	Frequency	PRR	IC	TSS	Signal detection			Labeling in MFDS
					PRR	IC	TSS	
**Unlabeled signalsfor cell-based vaccine compared to egg-based vaccine**								
**Application site disorders**^†^								
Injection site bruising	17	3.79	0.78	0.011	Y	Y	Y	N
**Respiratory system disorders**^†^								
Pneumonitis	3	22.75	0.84	0.388	Y	Y		N
**Central & peripheral nervous system disorders**^†^								
Encephalopathy	3	3.79	0.07		Y	Y		N
**Heart rate and rhythm disorders**^†^								
Tachycardia	3	7.58	0.49	0.952	Y	Y		N
**Unlabeled signals for egg-based vaccine compared to cell-based vaccine**								
**Body as a whole—general disorders**^†^								
Leg pain	73	4.81	0.33		Y			N
Tremor	51			0.018				N
**Application site disorders**^†^								
Injection site mass	110	2.90	0.27		Y			N
**Metabolic and nutritional disorders**^†^								
Cachexia	81	3.56	0.32		Y			N

Abbreviations: WHO-ART, World Health Organization-Adverse Reactions Terminology; PT, preferred term; AE, adverse events; IC, information component; PRR, proportional reporting ratio; TSS, tree-based scan statistic; MFDS, Ministry of Food and Drug Safety of South Korea.

^†^Adverse events were categorized according to the WHO-ART System Organ Class.

### Performance evaluation

**Figure 1:**
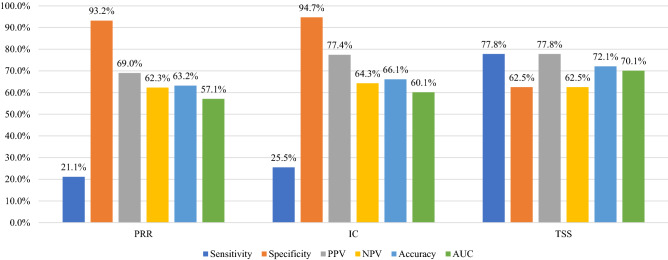
Bar chart of performance measurements for signal detection algorithms about influenza vaccine. Abbreviations: IC, information component; PRR, proportional reporting ratio; TSS, tree-based scan statistic; PPV, positive predictive value; NPV, negative predictive value; AUC, area under the curve.

We measured the performance indicators of each data mining method based on the confusion matrix, where all detected signals were categorized into true positive (TP), false positive (FP), true negative (TN), or false negative (FN) based on the reference standard. Overall, TSS demonstrated well balance. TSS showed the highest sensitivity (77.8%), positive predictive value (PPV, 77.8%), accuracy (72.1%), and area under the curve (AUC, 70.1%) and the lowest specificity (62.5%). In contrast, IC showed the highest specificity (94.7%) and negative predictive value (NPV, 64.3%). In addition, except for specificity, all PRR measurements were the lowest (Fig. 1).

## Discussion

We conducted a safety surveillance study on influenza vaccines and their subtypes using a nationwide spontaneous reporting database with three different data mining methods. The total number of signals detected by disproportionality-based analysis and TSS for the influenza vaccine compared to other vaccines were similar, and few unexpected AEs for the overall influenza vaccine were observed. Additional signal detection according to subtype was also conducted, where we noted that a wider range of AEs were detected as signals for TIVs and egg-based vaccines. Regarding the evaluation for signal detection, TSS showed better performance than the other data mining methods, with the optimal balance among all performance measurements.

From the results of the signal detection for the overall influenza vaccine, we identified that most signals were known AEs, such as application site disorders, body as a whole-general disorder, and musculoskeletal system disorders. All five serious AEs of special interest for influenza vaccine were detected as signals, except for febrile seizure. Similar findings were reported in two recent studies, both of which used two disproportionality-based methods (PRR and IC) for safety signal detection of influenza vaccines using the U.S. Vaccine Adverse Event Reporting System (VAERS), where GBS and paralysis were detected as signals by all three methods, respectively^20,21^. Among the seven detected signals not listed in the labeling in South Korea, injection-site inflammation and muscle weakness are reflected in the U.S. Food and Drug Administration (US-FDA) labeling. Accordingly, injection site inflammation and muscle weakness should be updated after signal evaluation. Of the remaining five unlabeled signals in both the U.S. and South Korea, narcolepsy seems to require urgent assessment. As mentioned earlier, narcolepsy cases grew rapidly following the 2009–2010 H1N1 vaccination in Finland and five other European countries (see Supplementary Material S5 online). Subsequent studies have shown that the AS03-adjuvanted pandemic vaccine Pandemrix (GlaxoSmithKline Biologicals, Wavre, Belgium), which was widely used in these countries during that period, contains a protein structurally similar to the orexin receptor, which is likely to induce an autoimmune response that destroys orexin cells in the brain and causes narcolepsy^22^. The H1N1 vaccine adjuvanted with AS03 was not used in Korea, but we identified narcolepsy as a safety signal for the influenza vaccine compared with other vaccines in this study, although there were only 11 reported cases. Therefore, signal validation through an in-depth evaluation of these cases is required.

Furthermore, we noted that the distribution of AEs differed between QIVs and TIVs. Local reactions at the injection site were more frequently reported in QIVs than in TIVs in our studies, which is consistent with previous studies. A meta-analysis of 5 RCTs reported that the incidence of injection site pain was higher with QIVs than TIVs with a pooled risk ratio of 1.18 (95% confidence interval: 1.03–1.35)^23^. In addition, a wider range of AEs were detected as signals for TIVs in our study, which showed inconsistent results with a previous RCT^24^ that demonstrated that QIVs and TIVs showed similar profiles. This may be attributed to the different distribution of age groups between QIVs and TIVs, with the proportion of the older group (65 years and above) being 0.47% and 10.1%, respectively. The aged population is vulnerable to adverse drug reactions due to age-related physiological changes^25^.

Moreover, we detected a wider range of AEs as signals with egg-based vaccines compared with cell-based vaccines, suggesting that safety profiles among these two subtypes may differ, which is inconsistent with the results of previous studies. Two RCTs demonstrated that cell-based and egg-based TIVs had comparable safety profiles in children and adolescents < 18 years of age^26,27^. We believe that this situation may be due to the following reasons. First, compared with cell-based vaccines, an inherent drawback of egg-based vaccines is that impurities or exogenous contamination may cause AEs^28^. In addition, Korea approved the first cell-based influenza vaccine in 2014, and egg-based influenza vaccines still dominate the majority of the market share^29^. In our database, cell-based vaccines only accounted for 15% of all cases; therefore, it is possible that some AEs of cell-based vaccines have not yet been revealed. Therefore, it is necessary to collect additional information.

In this study, TSS had the highest accuracy (72.1%), AUC (70.1%), PPV (77.8%), and sensitivity (77.8%). TSS allows the simultaneous evaluation of a large number of individual AE terms and related AE groups, thereby resolving multiple testing problems of disproportionality-based methods^30^. Two previous studies of the BCG vaccine and pneumococcal vaccine using the KAERS database also showed balanced performance in TSS^17,18^. However, we found that the sensitivity of TSS in this study was much higher than that in the BCG and pneumococcal vaccine studies (35.3% and 31.7%, respectively). There are two possible explanations for this observation. First, safety profiles are relatively well-established in the influenza vaccine, which targets the entire population as more extensive AEs have been reported and studied, compared to BCG and pneumococcal vaccines, both of which have limited recipients. Furthermore, the ability to detect signals through data mining techniques is largely influenced by the size of the dataset, which differs significantly between influenza and other vaccines (current study, 17,378 AE reports; BCG, 833 AE reports; pneumococcal vaccine, 1135 AE reports).

This study had several limitations. First, quantitative signal detection using passive surveillance systems, such as the KAERS database, has the inherent limitations of missing information, inconsistent quality of individual case safety reports, duplicated reporting, and under-reporting due to lack of awareness^31^. Therefore, our results do not reflect a causal relationship between vaccines and AEs, and further signal validation and assessment are needed. Second, in this study, the reference standard was established based on Ministry of Food and Drug Safety of South Korea (MFDS)-approved labeling for performance evaluation; thus, the results could be largely affected by the reference standard^32^. However, we believe that the labeling included all related AEs since influenza vaccines have been used for a long time. Third, our findings should not be overstated that TSS is the best-balanced method to detect safety signals in other vaccines or even in drugs as individual vaccine or drugs has different medical or clinical context. Therefore, further studies should be conducted to confirm our finding on the performance TSS across different vaccines and drugs. Lastly, although TSS showed the well-balanced performance compared to other data mining methods, TSS did not show the best values in all performance measurements, particularly in specificity. This result could be explained by the inverse relationship between sensitivity and specificity when considering the highest sensitivity in TSS, which were about threefold higher compared with the other methods (TSS: 77.8%, PRR: 21.1%, IC: 25.5%). This result suggests that the utilization of multiple data mining methods is reasonable choice to detect safety signals conservatively. In addition, the low values of accuracy and AUC were observed across all methods (TSS: 72.1%, 70.1%; PRR: 63.2%, 57.1%; IC: 66.1%, 60.1%). These results might be influenced by the reference standard that was used for constructing a confusion matrix for performance measurement. However, as the bias from reference standard could affect the performance of all methods, we believe that this bias is unlikely to distort the results of performance. Another possibility is that these methods are not precise or advanced to detect safety signals appropriately. Recently, machine learning algorithms have been utilized in the pharmacovigilance field with relatively high performance, compared with the traditional data mining methods^33^. However, we did not utilize this method in this study. Therefore, further research involving a variety of new methods such as machine learning is needed.

## Conclusions

In this post-marketing surveillance study, we identify 7 new safety signals for influenza vaccine including narcolepsy as a serious AE, and observed TIVs and egg-based influenza vaccine showed a wide range of new safety signals when compared to QIVs and cell-based influenza vaccine, respectively. Further studies are needed to confirm the potential relationship between influenza vaccine and new safety signals, and meanwhile, healthcare providers should evaluate whether vaccinees are susceptible to experiencing these safety signals by an in-depth review of comorbidities or AE-related history to prevent an avoidable risk. In addition, although TSS showed balanced performance in this study, there is still discrepancy in the identified safety signals among different methods, and some measurements were lower than the others. This result suggests that the complementary use of multiple data mining methods should be utilized to detect new safety signals conservatively.

## Methods

### Data source

We used the Korea Institute of Drug Safety and Risk Management-Korea Adverse Event Reporting System Database (KIDS-KAERS database)^34^ from 2005 to 2019. This database includes all spontaneous AE reports in South Korea and detailed information on demographics, vaccination, AEs, causality assessment, and clinical information. Information on the vaccine product was also provided. All drugs and AEs are coded according to the Anatomical Therapeutic Chemical Classification (ATC) system and the World Health Organization-Adverse Reaction Terminology (WHO-ART) dictionary^35,36^, respectively. WHO-ART is established as a tree structure, including system organ class (SOC), high-level terms (HLT), PTs, and included terms (IT) from top to bottom, and the signals were determined at the PT levels in this study.

The need for informed consent was waived, as this study was conducted using a spontaneous reporting database which does not include personal information. This study was approved by the Institutional Review Board of Chung-Ang University in South Korea (1041078-202008-ZZ-209-01). The Institutional Review Board of Chung-Ang University has waived informed consent for this study. All methods were carried out in accordance with the relevant guidelines and regulations.

### Study vaccine

All vaccines available in Korea were included. The study vaccine was an influenza vaccine, and all other vaccines were used as comparators for signal detection. Nineteen influenza vaccine products have been approved and used in Korea, with 2 cell-based vaccines, 17 egg-based vaccines, 8 TIVs, and 11 QIVs. A full list of influenza vaccine products used in our study is given in Supplementary Material S1 online.

### Selection of AE reports

From all AEs following vaccination collected in our database from 2005 to 2019, we excluded the following: (1) those that were reported as a concomitant vaccine, not a suspected vaccine; (2) those that were not final reports; (3) those that were invalid reports (missing or unspecified drugs or AE codes), and (4) those with report errors (logical error).

**Figure 2:**
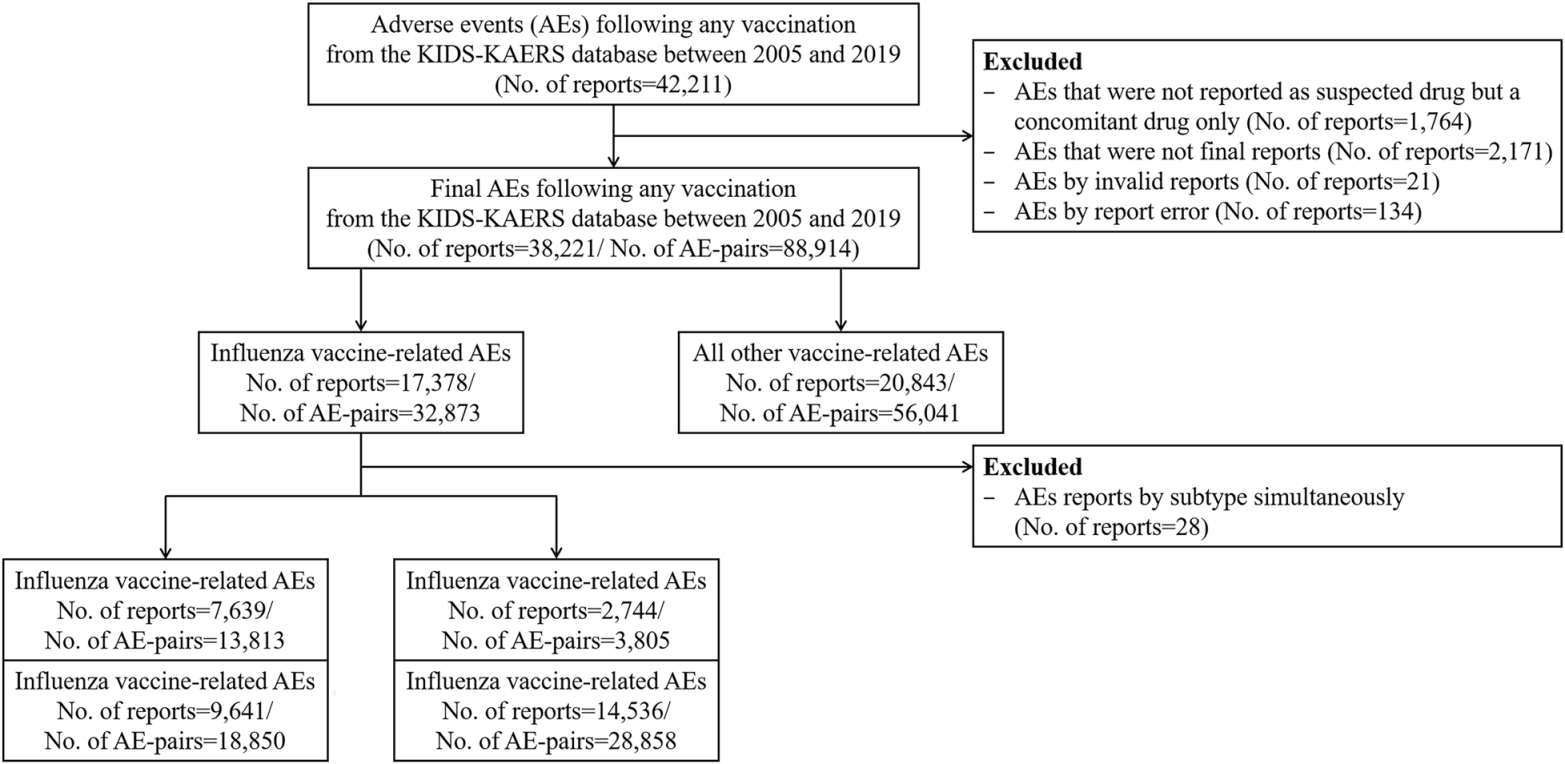
Flowchart of selection for spontaneous reports of adverse event. Abbreviations: KIDS-KAERS, Korea Institute of Drug Safety and Risk Management-Korea Adverse Event Reporting System Database; QIV, quadrivalent influenza vaccine; TIV, trivalent influenza vaccine. AE, adverse event.

From a total of 42,211 reports in our database, we identified 38,221 reports after vaccination. Among these, 17,378 (45.5%) were reported for the influenza vaccine. We identified 17,280 reports, of which 7639 (44.2%) were reported for QIV, 9641 (55.8%) for TIV, 2744 (15.9%) for cell-based influenza vaccine, and 14,536 (84.1%) for egg-based influenza vaccine. A flowchart of the study is presented in Fig. 2.

### Selection of five AEs of special interest

Based on literature REVIEWS, we selected the following five possible serious and important AEs observed after influenza vaccination: GBS, febrile conversion, anaphylaxis, narcolepsy, and Bell’s palsy. Detailed information is provided in Supplementary Material S5 online.

### Statistical analyses

#### Descriptive analyses

We calculated the frequency and proportion of baseline demographic characteristics (e.g., sex and age group) and reports (e.g., report type, source, reporter, seriousness, and causality assessment). Chi-squared tests were performed to compare the distribution of baseline characteristics between the influenza vaccine and all other vaccines or among individual subtypes of influenza vaccine (TIVs vs. QIVs and egg-based vs. cell-based vaccines).

#### Algorithms for signal detection

To detect the signals, three signal detection algorithms were used in this study, including PRR, IC, and TSS.

#### Disproportionality-based analysis

In this study, we used PRR and IC to generate signal scores for all AE pairs for the influenza vaccine versus all other vaccines, QIVs versus TIVs, and cell-based versus egg-based influenza vaccine. The Medicines and Healthcare Products Regulatory Agency (MHRA) in the United Kingdom and the WHO use PRR and IC, respectively^37^. The thresholds of signals for each algorithm were defined as follows: PRR ≥ 2, chi-squared test ≥ 4, number of reported AEs ≥ 3, IC with a lower limit of 95% confidence interval ≥ 0.

#### Tree-based scan statistic (TSS)

TSS is based on log-likelihood ratio (LLR) statistics and is suitable for analyzing a hierarchical structure variable. In this study, a diagnosis tree defined by the WHO-ART hierarchical structure was constructed, and the signals were determined at the PT level to compare them with the signals detected by the other algorithms. The unconditional Bernoulli model was selected to compare the number of AE reports between the two drugs. The expected and observed counts at each leaf were generated under the null hypothesis of an independent relationship between the vaccine and AE. AEs were defined as signals when the p-value of LLR was lower than 0.05, and p-values were generated using a Monte Carlo simulation. The analysis was performed using TreeScan Software^38^.

#### Performance evaluation of signal detection algorithms

The performance of disproportionality analysis (PRR, IC) and TSS was evaluated based on performance indicators such as sensitivity, specificity, PPV, NPV, accuracy, and AUC to identify which algorithm was more appropriate for detecting safety signals for influenza vaccines. To evaluate the performance indicators, a reference standard was established based on the MFDS-approved package inserts downloaded on September 13, 2020, from the NeDRUG website of the MFDS^39^.

## Supplementary Information

Supplementary Information.

## Data Availability

The data underlying this study are from the KAERS which has been transferred to the KIDS. The Korean government prohibits the release of the spontaneous reporting dataset to the public domain. Interested researchers can obtain the data through formal application to the KIDS. Korea Institute of Drug Safety & Risk Management (Ministry of Food and Drug Safety), Korean (https://open.drugsafe.or.kr/original/invitation.jsp).
