# Supplementary material for: Post-marketing surveillance study on influenza vaccine in South Korea using a nationwide spontaneous reporting database with multiple data mining methods
Source: Sci Rep. 2022 Nov 24;12:20256. doi: 10.1038/s41598-022-21986-8 (PMC9691710; doi:10.1038/s41598-022-21986-8)
Supplement: Supplementary file 1 — Supplementary Information. [file 41598_2022_21986_MOESM1_ESM.pdf]

Supplementary Material S1. All influenza vaccines available in South Korea

Vaccine	Approved age	Type	Product name
**TIVs**	6 months old or older	Egg-based	GCFlu Prefilled syringe inj, Boryung Fluvaccine V inj. (prefilled syringe), Boryung Fluvaccine VIII-TF® inj. (prefilled syringe), Vaxigrip, Flu plus TF inj., Il-Yang Fluvaccine prefilled syringe inj., Kovax influ PF inj. (prefilled syringe)
		Cell-based	SKYcellflu prefilled syringe
**QIVs**	6 months old or older	Egg-based	GCFlu quadrivalent prefilled syringe inj., Boryung FluV tetravaccine inj. (prefilled syringe), Boryung FluVIII tetravaccine inj. (prefilled syringe), Kovax flu quadrivalent PF inj. (prefilled syringe), BR Flutec tetravaccine inj. (prefilled syringe), Fluarix tetra prefilled syringe, Vaxigrip tetra inj.
	3 years old or older	Egg-based	Vaxiflu quadrivalent inj. (prefilled syringe), Kovax influ quadrivalent PF inj., Teratect prefilled syringe inj.
		Cell-based	SKYcellflu quadrivalent prefilled syringe

Abbreviations: QIV: quadrivalent influenza vaccines; TIV: trivalent influenza vaccine.

Supplementary Material S2. Signal detection results of the influenza vaccine using disproportionality methods and tree-based scan statistics from 2005 to 2019

Adverse events (WHO-ART PT code)	Frequency	PRR	IC	TSS	Signal detection			Labeling in MFDS
					PRR	IC	TSS	
**Application site disorders†**								
Injection site pain	7,291	2.71	0.69	0.001	Y	Y	Y	Y
Injection site inflammation	1,429	2.99	0.69	0.001	Y	Y	Y	N
Injection site pressure sensation	628	118.96	1.25	0.001	Y	Y	Y	Y
Infusion site rash	9	15.34	0.04	0.109	Y	Y		Y
**Body as a whole - general disorders†**								
Fatigue	1,640	12.05	1.15	0.001	Y	Y	Y	Y
Rigors	812	3.44	0.73	0.001	Y	Y	Y	Y
Malaise	651	4.91	0.86	0.001	Y	Y	Y	Y
Pain	628	2.12	0.44	0.001	Y	Y	Y	Y
Asthenia	466	3.89	0.74	0.001	Y	Y	Y	Y
Tenderness NOS	281	6.22	0.86	0.001	Y	Y	Y	Y
Chest pain	95	2.22	0.25	0.001	Y	Y	Y	Y
Anaphylactic reaction	39	2.77	0.18	0.01	Y	Y	Y	Y
**Musculo-skeletal system disorders†**								
Myalgia	3,079	3.74	0.83	0.001	Y	Y	Y	Y
Arthralgia	262	1.75	0.24	0.001		Y	Y	Y
Muscle weakness	111	3.32	0.49	0.001	Y	Y	Y	N
**Central & peripheral nervous system disorders†**								
Headache	1,544	3.81	0.81	0.001	Y	Y	Y	Y
Dizziness	548	1.48	0.18	0.001		Y	Y	Y
Paralysis	107	1.96	0.20	0.002		Y	Y	Y
Neuropathy	108	0.73	-1.71	0.001			Y	Y
Neuritis	99	5.82	0.68	0.001	Y	Y	Y	Y
Dysesthesia	48	2.10	0.08	0.182	Y	Y		Y
Quadriplegia	32	5.46	0.39	0.001	Y	Y	Y	N
Paraplegia	24	2.92	0.05	0.266	Y	Y		N
**Gastro-intestinal system disorders†**								
Nausea	518	2.31	0.48	0.001	Y	Y	Y	Y
**Respiratory system disorders†**								
Pleural pain	221		0.27	0.001			Y	Y
Dyspnea	208	3.25	0.57	0.001	Y	Y	Y	Y
Hyperventilation	18	10.23	0.32	0.002	Y	Y	Y	N
**Skin and appendages disorders†**								
Sweating increased	177	5.59	0.77	0.001	Y	Y	Y	Y
**Metabolic and nutritional disorders†**								
Cachexia	84	4.21	0.54	0.001	Y	Y	Y	N
**Psychiatric disorders†**								
Narcolepsy	11	18.75	0.16	0.011	Y	Y	Y	N

Abbreviations: WHO-ART, World Health Organization-Adverse Reactions Terminology; PT, preferred term; AE, adverse events; IC, information component; PRR, proportional reporting ratio; TSS, tree-based scan statistic; MFDS, Ministry of Food and Drug Safety of South Korea; NOS, not otherwise specified.

[†]Adverse events were categorized according to the WHO-ART System Organ Class.

Supplementary Material S3. Signal detection results for quadrivalent and trivalent influenza vaccines using disproportionality methods and tree-based scan statistics from 2005 to 2019

Adverse events (WHO-ART PT code)	Frequency	PRR	IC	TSS	Signal detection			Labeling in MFDS
					PRR	IC	TSS	
**QIV comparing to TIV**								
**Application site disorders†**								
Injection site pain	5,094	3.16	0.67	0.001	Y	Y	Y	Y
Injection site reaction	749	2.56	0.49	0.001	Y	Y	Y	Y
Injection site rash	515	1.51	0.16	0.001		Y	Y	Y
Injection site pruritus	144	1.72	0.11	0.001		Y	Y	Y
Injection site bruising	38	3.99	0.22	0.001	Y	Y	Y	N
Application site edema	30	3.72	0.12	0.005	Y	Y	Y	Y
Application site reaction	29	6.60	0.27	0.001	Y	Y	Y	Y
**Musculo-skeletal system disorders†**								
Myalgia	1,638	1.56	0.25	0.001		Y	Y	Y
**Body as a whole - general disorders†**								
Fatigue	829	1.40	0.14	0.001		Y	Y	Y
Rigors	405	1.36	0.07	0.001		Y	Y	Y
**Psychiatric disorders†**								
Somnolence	168	12.07	0.78	0.001	Y	Y	Y	Y
**Metabolic and nutritional disorders†**								
Cachexia	53	2.33	0.09	0.016	Y	Y	Y	N
**Hearing and vestibular disorders†**								
Application site pain	12		1.13	0.001			Y	Y
**TIV comparing to QIV**								
**Body as a whole - general disorders†**								
Fever	1,250	1.86	0.21	0.001		Y	Y	Y
Headache	1,075	1.69	0.16	0.001		Y	Y	Y
Pain	612	34.50	0.60	0.001	Y	Y	Y	Y
Nausea	422	3.26	0.31	0.001	Y	Y	Y	Y
Asthenia	371	2.96	0.27	0.001	Y	Y	Y	Y
Urticaria	347	3.91	0.34	0.001	Y	Y	Y	Y
Vomiting	311	6.33	0.41	0.001	Y	Y	Y	Y
Allergy	18	13.19	0.21	0.022	Y		Y	Y
Allergic reaction	133	2.44	0.09	0.001	Y	Y	Y	Y
Anaphylactic reaction	30	3.14	0.20		Y			Y
Edema	118	9.61	0.32	0.001	Y	Y	Y	Y
Chest pain	86	7.88	0.24	0.001	Y	Y	Y	Y
Leg pain	71	13.01	0.24	0.001	Y	Y	Y	Y
Tremor	48	11.73	0.13	0.001	Y	Y	Y	N
Hyperpyrexia	40	7.33	0.04	0.001	Y	Y	Y	N
Syncope	36	8.79	0.02	0.001	Y	Y	Y	Y
Death	23			0.001			Y	N
Edema peripheral	19	2.79	0.39		Y			N
Edema mouth	13	4.76	0.46		Y			N
**Central & peripheral nervous system disorders†**								
Dizziness	395	1.89	0.13	0.001		Y	Y	Y

Paresthesia	165	8.64	0.37		Y	Y	Y	Y
Paralysis	91	4.17	0.15	0.001	Y	Y	Y	Y
Neuritis	84	8.79	0.25	0.001	Y	Y	Y	Y
Convulsions	51	12.46	0.15	0.001	Y	Y	Y	Y
Dysesthesia	43	6.30	0.04	0.001	Y	Y	Y	Y
Quadriplegia	31		0.07	0.001		Y	Y	N
Gait abnormal	31	7.57	0.04	0.001	Y		Y	N
Hypoesthesia	28	5.13	0.13	0.022	Y		Y	N
Dyskinesia	20			0.001			Y	Y
Paraplegia	24			0.001			Y	Y
Dysphonia	19	2.79	0.39		Y			N
Stupor	14	10.26	0.34		Y			N
**Respiratory system disorders**[†]								
Pharyngitis	488	1.44	0.03	0.001		Y	Y	Y
Bronchitis	285	1.55	0.01	0.001		Y	Y	Y
Coughing	249	1.83	0.07	0.001		Y	Y	Y
Pleural pain	221		0.52	0.001		Y	Y	Y
Dyspnea	189	9.23	0.39	0.001	Y	Y	Y	Y
Upper respiratory tract infection	156	2.86	0.16	0.001	Y	Y	Y	Y
Pneumonia	93	4.01	0.15	0.001	Y	Y	Y	N
Sinusitis	72	2.51	0.02	0.009	Y		Y	Y
Induration	48		0.21			Y		Y
Asthma	30	3.66	0.16		Y			Y
Hyperventilation	18			0.001			Y	N
Tracheitis	9	6.60	0.61		Y			N
**Skin and appendages disorders**[†]								
Rash erythematous	238	14.53	0.46	0.001	Y	Y	Y	N
Sweating increased	142	2.97	0.15	0.001	Y	Y	Y	Y
**Induration**	48		0.21	0.001	Y		Y	Y
Angioedema	20	7.33	0.21		Y			Y
Skin discoloration	12		0.03				Y	N
Bullous eruption	12	4.40	0.51		Y			N
**Application site disorders**[†]								
Injection site pressure sensation	626	229.36	0.63	0.001	Y	Y	Y	Y
Tenderness NOS	279	102.22	0.54	0.001	Y	Y	Y	Y
Injection site infection	17	3.11	0.41		Y			Y
**Gastro-intestinal system disorders**[†]								
Gastroenteritis	37	2.71	0.17		Y			N
Dyspepsia	31	2.27	0.28		Y			N
**Musculo-skeletal system disorders**[†]								
Muscle weakness	103	15.10	0.33	0.001	Y	Y	Y	N
**Resistance mechanism disorders**[†]								
Infection viral	30	3.66	0.16		Y			N
**Cardiovascular disorders**[†]								
Hypotension	20	14.66	0.15	0.009	Y		Y	N
**Hearing and vestibular disorders**[†]								
Otitis media	41	10.02	0.07	0.001	Y	Y	Y	Y

Deafness	9	6.60	0.61	Y	N
**Vision disorder**†					
Vision abnormal	16	3.91	0.39	Y	N
**Reproductive disorders, female**†					
Dysmenorrhea	12	8.79	0.43	Y	N

Abbreviations: WHO-ART, World Health Organization-Adverse Reactions Terminology; PT, preferred term; AE, adverse events; IC, information component; PRR, proportional reporting ratio; TSS, tree-based scan statistic; MFDS, Ministry of Food and Drug Safety of South Korea; NOS, not otherwise specified; QIV, quadrivalent influenza vaccine; TIV, trivalent influenza vaccine.

†Adverse events were categorized according to the WHO-ART System Organ Class.

Supplementary Material S4. Signal detection results of cell-based and egg-based influenza vaccines using disproportionality methods and tree-based scan statistics from 2005 to 2019

Adverse events (WHO-ART PT code)	Frequency	PRR	IC	TSS	Signal detection			Labeling in MFDS
					PRR	IC	TSS	
**Cell-based compared to egg-based vaccine**								
**Application site disorders**†								
Injection site pain	1,713	2.33	0.93	0.001	Y	Y	Y	Y
Injection site reaction	184	1.45	0.23	0.002		Y	Y	Y
Injection site bruising	17	3.79	0.78	0.011	Y	Y	Y	N
Application site edema	15	4.38	0.86	0.011	Y	Y	Y	Y
**Psychiatric disorders**†								
Somnolence	49	2.69	0.73	0.001	Y	Y	Y	Y
**Gastro-intestinal system disorders**†								
Enteritis	9	2.13	0.01		Y	Y		Y
**Respiratory system disorders**†								
Tracheitis	9	68.26	1.70	0.001	Y	Y	Y	Y
Pneumonitis	3	22.75	0.84	0.388	Y	Y		N
**Central & peripheral nervous system disorders**†								
Encephalopathy	3	3.79	0.07		Y	Y		N
Encephalomyelitis	2	7.58	0.26			Y		Y
Torticollis	2	15.17	0.48	0.985		Y		Y
**Heart rate and rhythm disorders**†								
Tachycardia	3	7.58	0.49	0.952	Y	Y		N
**Respiratory system disorders**†								
Stridor	2	7.58	0.26			Y		Y
**Egg-based compared to cell-based vaccine**								
**Body as a whole - general disorders**†								
Fever	1,608			0.001			Y	Y
Malaise	631	4.16	0.03	0.001	Y		Y	Y
Pain	624	82.28	0.01	0.001	Y	Y	Y	Y
Nausea	500	3.88	0.05	0.001	Y		Y	Y
Urticaria	389	2.23	0.11	0.002	Y		Y	Y
Vomiting	339	5.59	0.07	0.001	Y		Y	Y
Abdominal pain	135	2.97	0.23		Y			Y
Edema	121	2.66	0.26		Y			Y
Leg pain	73	4.81	0.33		Y			N
Tremor	51			0.018			Y	N
**Central & peripheral nervous system disorders**†								
Headache	1,439			0.001			Y	Y
Dizziness	512			0.003			Y	Y
Paresthesia	173	3.80	0.18	0.002	Y		Y	Y
Paralysis	106	13.98	0.23	0.002	Y		Y	Y
Dysesthesia	48			0.025			Y	Y
**Respiratory system disorders**†								
Pleural pain	221			0.001			Y	Y
Dyspnea	201	8.83	0.13	0.001	Y		Y	Y
Pneumonia	105	2.77	0.28		Y			Y

Chest pain	92	6.07	0.27	0.023	Y		Y	Y
Sinusitis	91	6.00	0.27	0.029	Y		Y	Y
Induration	48			0.025			Y	Y
**Skin and appendages disorders**†								
Rash erythematous	247	10.86	0.10	0.001	Y		Y	Y
Rash	220	2.07	0.18		Y			Y
Sweating increased	176	23.21	0.13	0.001	Y		Y	Y
**Application site disorders**†								
Injection site rash	911			0.001			Y	Y
Injection site pressure sensation	628		0.02	0.001		Y	Y	Y
Tenderness NOS	281			0.001			Y	Y
Injection site mass	110	2.90	0.27		Y			N
**Musculo-skeletal system disorders**†								
Arthralgia	249	2.74	0.14	0.004	Y		Y	Y
**Metabolic and nutritional disorders**†								
Cachexia	81	3.56	0.32		Y			N

Abbreviations: WHO-ART, World Health Organization-Adverse Reactions Terminology; PT, preferred term; AE, adverse events; IC, information component; PRR, proportional reporting ratio; TSS, tree-based scan statistic; MFDS, Ministry of Food and Drug Safety of South Korea; NOS, not otherwise specified.

†Adverse events were categorized according to the WHO-ART System Organ Class.

Supplementary Material S5. Detailed information of 5 special interest of AEs

1) Guillain–Barré Syndrome (GBS)

GBS is a rapid-onset muscle weakness caused by the immune system, which damages the peripheral nervous system. Following influenza vaccination, GBS was first reported in the US during the influenza season of 1976–1977, with an estimated population attributable risk (PAR) of 1 per 100,000 vaccinations [1]. Research conducted in Korea in 2015 revealed the incidence of GBS was 0.87 in the pandemic period (May 2009–April 2010) following the influenza vaccine, compared with 0.63 in an earlier reference period (May 2008–April 2009) [2].

2) Febrile seizure

Febrile seizure is a seizure associated with a high body temperature but without any serious underlying health issue and most commonly occurs in children between the ages of 6 months and 5 years [3]. There was a rapid increase in febrile seizure cases after influenza vaccination in Australia in 2010, with an estimated PAR of 3.3 per 1,000 vaccinations [4]. In addition, in the United States, the Vaccine Adverse Event Reporting System database (VAERS) reported 52 cases of 456 febrile convulsions in children under 5 years of age between 2010 and 2011.

3) Anaphylaxis

Anaphylaxis, often known as anaphylactic shock, is a serious allergic reaction that has a rapid onset and may cause death [5]. Several cases of anaphylaxis following influenza vaccination have been reported worldwide. After the 2009 H1N1 influenza vaccination, anaphylaxis was reported in 1 and 3.5 per 1,000,000 vaccinations in the U.S. [6] and Europe [7], respectively. In Korea, out of 13.8 million people who received influenza vaccines, 5 anaphylaxis cases and 10 anaphylactoid reactions have been reported [8].

4) Narcolepsy

Narcolepsy is a chronic neurological disorder that affects the capacity of the brain to govern

sleep-wake cycles. During the 2009-2010 H1N1 vaccination, a rapid increase in narcolepsy cases was reported in children and adolescents aged between 4 and 19 years in Finland, with PARs of 0.79 per 100,000 persons in 2009 and 9 per 100,000 persons in 2010 [9]. Subsequently, five other European countries (Sweden, France, England, Ireland, and Norway) reported an unusual increase in the number of narcolepsy cases during the 2009 H1N1 influenza pandemic. However, no narcolepsy cases have been reported in the U.S. [10] .

5) Bell's palsy

Bell's palsy (BP) is an unexplained episode of facial muscle weakness or paralysis. During the influenza season of 2000-2001, 46 cases of BP related to the influenza vaccine were reported in April 2001 [11]. In addition, out of 197 BP cases (94.2%) in the U.S. VAERS during 1991-2001, 145 were attributed to influenza vaccines [12].

REFERENCES

1. Schonberger, L.B., et al., *Guillain-Barre syndrome following vaccination in the National Influenza Immunization Program, United States, 1976--1977.* Am J Epidemiol, 1979. **110**(2): p. 105-23.
2. Kim, C., et al., *Pandemic influenza A vaccination and incidence of Guillain-Barré syndrome in Korea.* Vaccine, 2015. **33**(15): p. 1815-23.
3. Graves, R.C., K. Oehler, and L.E. Tingle, *Febrile seizures: risks, evaluation, and prognosis.* Am Fam Physician, 2012. **85**(2): p. 149-53.
4. Armstrong, P.K., et al., *Epidemiological study of severe febrile reactions in young children in Western Australia caused by a 2010 trivalent inactivated influenza vaccine.* BMJ Open, 2011. **1**(1): p. e000016.
5. Sampson, H.A., et al., *Second symposium on the definition and management of anaphylaxis: summary report--Second National Institute of Allergy and Infectious Disease/Food Allergy and Anaphylaxis Network symposium.* J Allergy Clin Immunol, 2006. **117**(2): p. 391-7.
6. Halsey, N.A., et al., *Immediate hypersensitivity reactions following monovalent 2009 pandemic influenza A (H1N1) vaccines: reports to VAERS.* Vaccine, 2013. **31**(51): p. 6107-12.
7. Banzhoff, A., S. Haertel, and M. Praus, *Passive surveillance of adverse events of an MF59-adjuvanted H1N1v vaccine during the pandemic mass vaccinations.* Hum Vaccin, 2011. **7**(5): p. 539-48.
8. Kim, J.H., et al., *Adverse events following immunization (AEFI) with the novel influenza a (H1N1) 2009 vaccine: findings from the national registry of all vaccine recipients and AEFI and the passive surveillance system in South Korea.* Jpn J Infect Dis, 2012. **65**(2): p. 99-104.

9. Nohynek, H., et al., *AS03 adjuvanted AH1N1 vaccine associated with an abrupt increase in the incidence of childhood narcolepsy in Finland.* PLoS One, 2012. **7**(3): p. e33536.

10. Sarkanen, T.O., et al., *Incidence of narcolepsy after H1N1 influenza and vaccinations: Systematic review and meta-analysis.* Sleep Med Rev, 2018. **38**: p. 177-186.

11. Mutsch, M., et al., *Use of the inactivated intranasal influenza vaccine and the risk of Bell's palsy in Switzerland.* N Engl J Med, 2004. **350**(9): p. 896-903.

12. Zhou, W., et al., *A potential signal of Bell's palsy after parenteral inactivated influenza vaccines: reports to the Vaccine Adverse Event Reporting System (VAERS)--United States, 1991-2001.* Pharmacoepidemiol Drug Saf, 2004. **13**(8): p. 505-10.
